# The extraordinary transformation of traditional Chinese medicine: processing with liquid excipients

**DOI:** 10.1080/13880209.2020.1778740

**Published:** 2020-07-02

**Authors:** Zhi Chen, Si-Yong Ye, Rong-Gang Zhu

**Affiliations:** a Pharmaceutical College, Shandong University of TCM, Jinan, China; b Department of Pharmacy, Jinan Second People’s Hospital, Jinan, China

**Keywords:** Pharmacological effects, synergism, chemical constitution

## Abstract

**Context:**

The Chinese medicinal materials originate from animals, plants, or minerals must undergo appropriate treatment before use as decoction pieces. Processing of Chinese medicines with liquid excipients is a pharmaceutical technique that transforms medicinal raw materials into decoction pieces which are significantly different from the original form. During processing, significant changes occur in chemical constituents, which inevitably affects clinical efficacy. At present, the liquid materials in processing mainly involve wine, vinegar, honey, saline water, ginger juice, herbal juice, etc.

**Objective:**

This review introduces the typical methods of liquid excipients processing, summarizes the influence on chemical composition, pharmacological efficacy, and expounds the ways and mechanisms of liquid excipients to change the properties of drugs, enhance the efficacy, eliminate or reduce toxicity and adverse reaction.

**Methods:**

English and Chinese literature from 1986 to 2020 was collected from databases including Web of Science, PubMed, Elsevier, Chinese Pharmacopoeia 2015, and CNKI (Chinese). Liquid excipients, processing, pharmacological effects, synergism, chemical constitution, traditional Chinese medicine (TCM) were used as the key words.

**Results:**

Liquid excipients play a key role in the application of TCM. Processing with proper liquid excipients can change the content of toxic or active components by physical or chemical transformation, decrease or increase drug dissolution, alter drug pharmacokinetics, or exert their own pharmacological effects. Thus, processing with liquid excipients is essential to ensure the safety and efficacy of TCM in clinic.

**Conclusion:**

This article could be helpful for researchers who are interested in traditional Chinese herbs processed with liquid excipients.

## Introduction

Traditional Chinese medicine (TCM) must be processed before it can be used in the clinic, and this is one of the characteristics of traditional Chinese medicine (Sheridan et al. 2015). Standardization of processing methods for TCM is as important as authentication to ensure their safe use and maintain their quality (Zhao et al. 2006). There are many ways to process, including: stir-frying, stir-frying without excipients, stir-frying with liquid excipients, stir-frying with solid excipients, steaming, boiling, stewing, etc. (Wu et al. 2018). Liquid excipients are widely used in the processing to minimize toxicity or enhance therapeutic effects and their history can be traced back to the Spring and Autumn periods (770–221 B.C.). After thousands of years of clinical practice, a variety of products processed with liquid excipients have been produced. According to TCM property theory, TCM has special affinities to certain organs and channel systems of the body, thus showing special effects on diseases of different systems and organs (Xu 2004). Liquid excipients can introduce herbs into different meridians according to the characteristics and clinical purpose of TCM. At present, the liquid materials in processing mainly involve wine, vinegar, honey, saline water, ginger juice, herbal juice, etc. Processing with wine can promote the upward direction and clean the upper-energizer heat; processing with vinegar adds to the liver-soothing and analgesic effects of drugs; honey confers Qi-nourishing and lung-moistening effects; processing with salt water introduces medicine into kidney, etc. (Li et al. 2016). Though classic processing theory and methods have been proven reasonable and reliable in the long-standing clinical practice, the underlying scientific principles remain largely unknown, affecting the production and use of decoction pieces.

**Figure 1.**
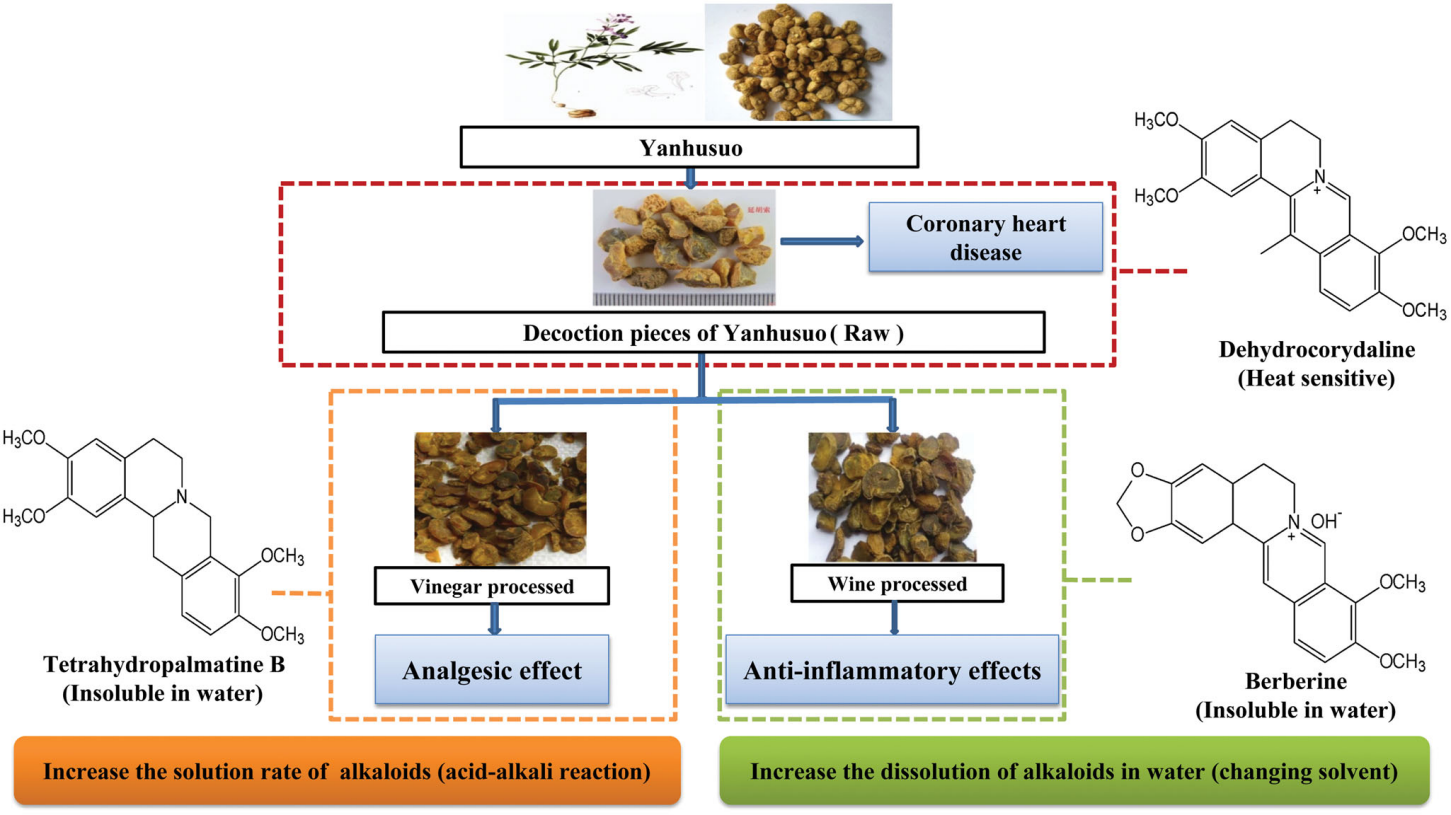
Different effects of *Corydalis yanhusuo* W.T. Wang (Paraveraceae) tuber (Yanhusuo) subjected to various processing methods.

The change of TCM properties is a core processing principle of Chinese herbal medicine, its origin being the material base and bioactivity change. Even in the same herb processed with different liquid excipients, the chemical composition and content of the drug will vary greatly and affect the clinical efficacy. *Corydalis yanhusuo* W.T.Wang. (Papaveraceae) tuber (Yanhusuo) has the function of moving ‘Qi’, activating blood and relieving pain. It is used as an herbal medicine for the treatment of heart pain, impediments, dysmenorrhoea, amenorrhoea, obstruction and postpartum stasis (Tian et al. 2020). Current research (Wang et al. 2011; Zhang et al. 2020) mainly focuses on tertiary and quaternary alkaloids which are considered as the effective components of Yanhusuo. According to the Chinese Pharmacopoeia Commission (2015 edition), the quality maker for Yanhusuo is tetrahydropalmatine which is proven one of the main alkaloids responsible for the pharmacological properties (Chinese Pharmacopoeia Commission 2015). Crude drugs should be processed before using, Yanhusuo has been used mainly in rice vinegar and yellow rice wine processed form. Rice vinegar improve the pain relief effect, while yellow rice wine increases the efficacy of the herbal drug (Bensky et al. 2004). The vinegar solubilizes the free alkaloids of Yanhusuo based on acid–base reaction, while the wine is a good organic solvent that can increase the water solubility of active substances. Researchers (Zhang et al. 2009; Dou et al. 2012) found processing with vinegar can improve the analgesic effect of Yanhusuo and can also change the pharmacokinetics of the main alkaloids. For example, vinegar processing was shown to cause a decrease in berberine and protopine and an increase in tetrahydropalmatine (Chen et al. 2003; Zhang et al. 2008). In clinical trials, vinegar processed Yanhusuo exhibited the best analgesic effect and has been used in the treatment of gynecological disorders especially in dysmenorrhoea. Wine processed Yanhusuo caused the dissolution of total alkaloids in water, which mainly plays a role in antithrombotic, antinociceptive and antiinflammatory effects (Li et al. 2013a). The different effects of different processed Yanhusuo are shown in Figure 1.

Compared to the raw decoction, processed decoction with proper liquid excipients can play a better role in disease prevention and treatment and this has been proven reliable and reasonable in the long clinical practice of TCM. However, this method also complicates the components of TCM. The chemical components of crude drugs may change dramatically during processing. In some cases, the change of chemical components is consistent with the purpose of processing with liquid excipients and can be verified by the modern research results (Zhong et al. 2019a). But in some cases, the processing theory remains to be explored. Meanwhile, the source and quality of liquid excipients notably affect the efficacy of processed drugs. The chemical components of raw decoction and processed decoction differ: new components may be formed or the relative contents may change; other components may disappear or their contents may decrease (Liu et al. 2020). Thus, the processing with liquid excipients needs to be further organized, validated and implemented with scientific understanding to safeguard the quality of TCM. Significant progress made in this direction in the recent years necessitates a systematic review to summarize the accumulated knowledge. This review summarizes the history and various processing methods with liquid excipients and highlights the possible material bases for the changing before and after processing in recent years.

## Development history

As early as Spring and Autumn period (770–221 B.C.), some Chinese doctors knew and recorded the processing methods with liquid excipients of some drugs. For example, *Phytolacca acinosa* Roxb. (Phytolaecaceae) root (Shanglu) was processed with vinegar and pills were made with wine in this period. The original drug processing technology with liquid excipients was described in the Handbook of Prescriptions for Emergencies written during the Eastern Jin Dynasty. With constant development, the drug processing technology with liquid excipients made great progress during the Han Dynasty including *Pinellia ternata* (Thunb.) Breit. (Araceae) tuber (Banxia), washed with wine, and *Aconitum carmichaeli* Debx. (Polygonaceae) maternal root (Heshouwu), processed with honey. During the Northern and Southern Dynasties, there were more and more drugs processed with liquid excipients and processing methods became more detailed. By the Tang Dynasty, TCM processing developed rapidly with increasing methods. The most common liquid processing methods included drugs processed with urine of boys under twelve and Heshouwu steamed with black soya beans. People paid more attention to processing excipients and systematic theory of processing with excipients appeared by the Yuan and Ming Dynasties (1271–1644). In the Qing Dynasty (1636–1912), the processing theory was developed mainly because people drew lessons from clinical experience. Through long-term clinical practice by TCM physicians, processing with liquid excipients has been developed and many of those methods are still being used today. There are 117 kinds of decoction pieces that are processed with various excipients, accounting for 55% of the total number of listed drugs in Chinese Pharmacopoeia Commission (2015 edition). The commonly used liquid adjuvants during processing are described below and listed in Table 1.

**Table 1.** Typical processing with liquid excipients and representative processed traditional Chinese herbal medicines listed in CP (2015 edition).

Processing with liquid excipients	Kind of liquid excipient	Processing method (Classification)	Traditional theory	Properties of drugs	Representative drugs
Processing with wine	1. Yellow rice wine 2. Chinese spirits	Stir-frying Stewing Quenching Steaming	Leading drugs upward	Drugs of bitter and cold	*Coptis chinensis* Franch. (Ranunculaceae) root (Huanglian)
				Drugs for promoting blood circulation and removing blood stasis	*Salvia miltiorrhiza* Bunge. (Lamiaceae) root and rhizome (Danshen)
				Tonic drugs	*Rehmannia glutinosa* (Gaert.) Libosch. ex Fisch. et Mey. (Scrophulariaceae) tuberous root (Dihuang)
				Smelly drugs	*Zaocys dhumnades*(Cantor). (Colubridae) body (Wushaoshe)
Processing with vinegar	1. Rice vinegar 2. Sorghum vinegar	Stir-frying Boiling Stewing Quenching Purficating	Introducing medicine into live	Drugs for removing stagnation	*Curcuma aromatica* Salisb. (Zingiberaceae) tuberous root (Yujin)
				Drastically purgating water drug	*Euphorbia kansui* T. N. Liou ex S. B. Ho. (Euphorbiaceae) tuberous root (Gansui)
				Drugs for arresting discharge	*Schisandra chinensis* (Turcz.) Baill. (Magnoliaceae) fructus (Wuweizi)
				Smelly drugs	*Boswellia bhaurdajiana* Birdw. (Burseraceae) resin (Ruxiang)
	
Processing with honey	Refined honey	Stir-frying Baking	Alleviating property and moistening lung	Drugs for relieving cough and asthma	*Tussilago farfara Linn*. (Compsitea) flowers (Kuandonghua)
				Drugs for tonifying middle-Jiao and Qi	*Glycyrrhiza uralensis* Fisch. (Leguminosae) root and rhizome (Gancao)
				Drugs for relieving exterior disorder	*Ephedra sinica* Stapf. (Ephedraceae) herbaceous stem (Mahuang)
				Drugs with toxic and side effects	*Aristolochia debilis* Sieb. et Zucc. (Aristolochiaceae) fructus (Madouling)
Processing with salt water	Salt water	Stir-frying Steaming	Introducing medicine into kidney	Kidney-reinforcing drugs	*Eucommia ulmoides* Oliver. (Eucommiaceae) bark (Duzhong)
				Drugs for securing essence and reducing urination	*Alpinia oxyphylla* Miq. (Zingiberaceae) fructus (Yizhiren)
				Heat-clearing drugs	*Anemarrhena asphodeloides* Bunge. (Liliaceae) root and rhizome (Zhimu)
Processing with ginger juice	1. Fresh ginger juice 2. Dried ginger juice	Stir-frying Boiling	Diffusing	Drugs of bitter and cold	*Coptis chinensis* Franch. (Ranunculaceae) root (Huanglian)
				Drugs for removing phlegm and relieving vomitin	*Caulis bambusae* in Taenia (Zhuru)
				Drugs with side effect	*Aristolochia debilis* Sieb. et Zucc. (Aristolochiaceae) seed (Madouling)
Processing with oil	1. Plant oil 2. Tallow	Stir-frying Frying Baking	Increasing effect and decreasing toxicity	Drugs for warming kidney and enhancing yang	*Epimedium brevicornu* Maxim. (Berberidaceae) leaf (Yinyanghuo)
				Toxic drugs	*Strychnos nux-vomica* Linn. (Loganiaceae) seed (Maqianzi)
				Drugs of hard texture	*Panax pseudo-ginseng* Wall. var. notoginseng (Burkill)Hoo & Tseng. (Araliaceae) root and rhizome (Sanqi)
Processing with bile	Bile	Fermentation	Changing the properties	Bitter and cold drug	*Arisaema heterophyllum* Blume. (Araceae) tuber (Tiannanxing)
Processing with herbal extract	1. Gancao extract 2. Black soya bean juice	Boiling Steaming	Synergism and attenuation	Drugs with side effect	*Polygala tenuifolia* Willd. (Polygalaceae) root (Yuanzhi)
				Hepatic and kidney-tonifying drug	*Fallopia multiflora* (Thunb.) Harald. (Polygonaceae) tuberous root (Heshouwu)

## Processing with different liquid excipients

### Processing with wine

**Figure 2.**
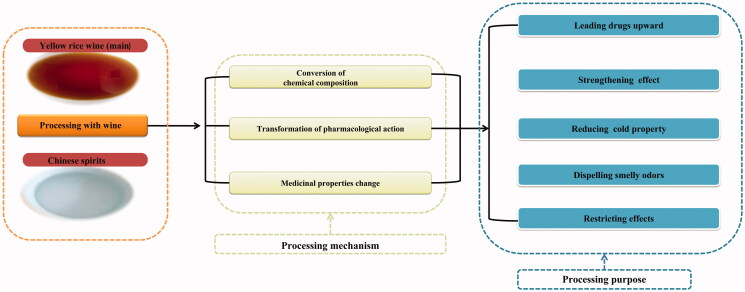
Processing mechanism and processing purpose with wine.

Processing of TCM with wine means to infiltrate crude drugs with a certain amount of wine used for processing or to process sliced drugs by approaches, such as stir-frying, stewing, steaming, tempering and quenching according to TCM theory. According to ancient codes and records during the Yuan Dynasty, ‘if a disease attacks people’s head, face and surface of limb muscle, the drugs should be stir-fried with wine to ascend via the power of wine’, indicating wide and important application of wine as excipients to drug processing (Wang 2011). Yellow rice wine and Chinese spirit are two major types of wine used for drug processing. Compared with yellow rice wine, Chinese spirit has a short history in processing. Yellow rice wine is the first choice in most cases (Li et al. 2013b). Researchers (Lu et al. 2015; Cai et al. 2019; Yu et al. 2019) found that yellow rice wine contains potentially bioactive components and has great antioxidant activity. It can enter the blood system, promote circulation of both blood and Qi and harmonize the blood (Li et al. 2013c). Chinese spirit is thick in Qi and can enter the Qi system, remove accumulated coldness, dispel cold and dissolve stagnant Qi. The alcohol content of yellow rice wine used for drug processing should meet the standard and generally ranges between 10 and 20%. The wine should be transparent without sediment impurities and not be fermented or smelly. Traditionally, wine is widely used for processing drugs with bitter cold, blood activating, tonic properties and drugs of animal origin. A diagram of general effects of processing with wine is shown in Figure 2.

Wine can strengthen the effects of invigorating the blood and promoting the upward direction. Both yellow rice wine and Chinese spirit contain alcohol, which can excite heart, promote the blood circulation and pass through membranous structure easily. These effects are consistent with the opinion that wine is good at promoting the blood circulation in TCM. In addition, processing with wine is accompanied with dissolution, displacement, diffusion and digestion of chemical constituents. As alcohol is a polar and nonpolar solvent, it promotes dissolution and has effects of desorption and adsorption to promote digestion and extraction of chemical constituents. After oral administration of drugs, alcohol allows more active substances enter the blood circulation, including big fat-soluble substances, so that it plays a role in ‘promoting the drug efficacy’ and ‘ascending the drug efficacy’. *Angelica sinensis* (Oliv.) Diels. (Umbelliferae) root (Danggui) can enrich the blood and invigorate blood circulation. After stir-frying of Danggui with wine, its effect of invigorating the circulation of blood is strengthened. *Scutellaria baicalensis* Georgi. (Labiatae) root (Huangqin) is a well-known TCM used for the treatment of jaundice, pyrexia inflammation, etc. It can be applied to clearing of upper-jiao heat after processing with wine. Flavonoids in Huangqin play an important role in metabolism and pharmaceutical effect, so they have become the focus of research (Sun et al. 2020). Researchers (Huang et al. 2014) found the pharmacokinetic parameters of C_max_ and AUC_0–t_ of some flavonoids in wine-processed Huangqin were remarkably increased in the rat upper-energizer tissues (heart and lung) but significantly decreased in the rat middle- and lower-energizer tissues (liver, spleen and kidney). Tissue distribution of flavonoids might be helpful in explaining the effects of wine-processing on ascending theory. The main mechanism of heat treatment in wine processing on flavonoids of Huangqin was mainly attributed of total surface area, fractal dimension and mesopores (Zhang et al. 2016).

Due to the wine′s warm, wine processing can reduce the cold property and enhance the tonic effect of the drugs. For instance, as *Rheum nobile* Hook.f. et Thoms. (Polygonaceae) root (Dahuang) and Huanglian are bitter and cold in nature, they can do harm to yang of spleen and stomach (Qin et al. 2017). However, the cold property of these drugs can be relieved (Huang et al. 2001) and the anti-blood stasis effects can be strengthened after processing with wine (Wei et al. 2019). Dahuang processing with wine involves two processes, namely heating and immersion in wine. Hence, influences of processing with wine on physicochemical properties of drugs are affected by these two aspects. Heat treatment decomposes the conjugated anthraquinones, and wine can affect distribution and change of chemical components, thereby enhancing the efficacy and reducing the toxicity of Dahuang. *Rehmannia glutinosa* (Gaert) Libosch. ex Fisch. et Mey. (Scrpophulariaceae) tuberous root (Dihuang) is commonly used as natural medicine singly or combination with other herbs to treat skeletal diseases (Liu et al. 2017) for thousands of years and has been used with increasing frequency. It contains polysaccharide, oestrogenic, catalpol, etc. (Tan et al. 2012; Lai et al. 2015). By comparing the chemical composition, the most recent report (Gong et al. 2019) found that iridoid glycosides were decreased and furfural derivatives increased in Dihuang after processing with wine which may be the chemical mechanism contribute to the differences in efficacy. *Cornus officinalis* Sieb. & Zucc. (Cornaceae) fructus (Shanzhuyu), a food and medicinal plant in China, contains 16 amino acids and a large number of essential elements for the human body, may be used as a valuable food supplement for the treatment of diabetes mellitus and have protective effects on osteoporosis (He et al. 2016; Yue et al. 2018; Sun et al. 2019). After the wine steaming of Shanzhuyu, the contents of loganin and morroniside are decreased, while ursolic acid and oleanolic acid contents are increased. It is consistent with the theory of TCM that raw Shanzhuyu is used for solid sweating and wine-processed products are used for tonifying liver and kidney (Liu 2019). Wine also can form crystalloid molecular compounds with inorganic constituents of plants to dissolve more inorganic constituents out. After processing with wine, 15 of Shanzhuyu’s 18 kinds of inorganic elements increased to varying degrees (Ding et al. 2007).

Moreover, wine processing can dispel smelly odours and loosen the texture for easy crushing and extracting. The common smelly odours of animal drugs make it difficult for patients to take and it mainly contains three aspects: protein cleavage, fatty acid oxidation, trimethylamine decomposition. Wine processing can reduce the content of this gut microbial metabolite of choline effectively. *Bungarus multicinctus.* Blyth. (Viperidae) body (Baihuashe) and *Zaocys dhumnades.,* Cantor. (Colubridae) body (Wushe) can remove their fishy smell with wine. However, this method is not suitable for drugs containing heat-sensitive ingredients, and whether it will reduce the effect of protein and amino acid also need to be studied. Mineral drug, such as actynolin, is hard, it must be processed by calcining and quenching with wine. Though some processed drugs contain no wine, the action of processing with wine should not explained by the alcohol content of a processed drug only. Moreover, we should not judge quality of processed drugs or processing with wine according to wine content of these drugs.

### Processing with vinegar

Processing of TCM with vinegar is a traditional processing technique, where crude drugs or medicine material crude slices are mixed with vinegar uniformly to deeply infiltrate vinegar into drugs and then heat treatment by stir-frying, stewing, quenching and steaming etc., are carried out. According to Correlation between Materia Medica Companion (MMC) written during the Ming Dynasty, ‘pain can be relieved by using drugs processed with vinegar for the liver meridian’, indicating that rice vinegar can play a medical role in leading drugs to affected parts with high targeting selectivity (Chen 1988). Thus, it can promote the therapeutic effect of drugs to some extent.

Vinegar has been used as an acid condiment for thousands of years (Mas et al. 2014). Chinese vinegar is mainly produced by typical solid-state fermentation techniques by grain (Nie et al. 2013). It is rich in protein, organic acid, sugar, amino acids, other flavour ingredients, and other nutrients (Budak et al. 2014; Chen et al. 2016). Processing with vinegar allows constituents to volatilized as the temperature rises and dissolution increased of active ingredients through changing the solvent, which alleviates the ascending property of drugs and reinforces effects of dispersing the liver, dissipating blood stasis and relieving pain. After processing, chemical components of drugs changed; active constituents increased and toxic constituents decreased, for example. This method is often used to process drugs of dispersing liver and relieving depression, eliminating stasis to stop pain and expelling water by purgation.

Drug processing with vinegar can lead drugs to the liver meridian and strengthening the effect of dispersing the depressed liver-Qi. *Bupleurum chinensis* DC. (Umbelliferae) root (Chaihu) has been used in TCM for over 2000 years with functions of clearing heat, reliving exterior syndrome and regulating liver-qi (Yuan et al. 2017a). Saikosaponins and volatile oils are considered to be the main ingredient of this drug. After processing with vinegar, its effects of dispersing the depressed liver-Qi are reinforced (Lei et al. 2018). In the meantime, the effects of anti-inflammation and reducing fever showed a significant trend. Modern research has found that the reason is due to a combination of heat action and acetic acid in vinegar. With this approach, the contents of volatile oils and anti-inflammatory components have substantially declined, while the content of saikosaponins B significantly increased. Saikosaponins B has the liver protective effect and modern pharmacological experiments have proven that processed Chaihu exhibited better liver protective effect than the raw decoction (Li et al. 2015). By clinical practice of TCM, it has been found that effects of invigorating the circulation of blood, reinforcing Qi and relieving pain of Yanhusuo are obviously strengthened after processing with vinegar. Modern research also shows that acetic acid can be combined with alkaloid in Yanhusuo to generate acetate, which reinforces its solubility in water (Tao et al. 2019). Hence, the analgesic effect of Yanhusuo processed with vinegar is better than that of crude drugs. *Schisandra chinensis* (Turca). Baill. (Magnoliaceae) fructus (Wuweizi) is sour in taste and astringent in nature and processing with vinegar can strengthen its astringent effect and inhibitory effect (Su et al. 2013). *Curcuma zedoaria* (Chiristm.) Rosc. (Zingiberaceae) root and rhizome (Ezhu) has pharmacological effects against cancer, fibrosis, cardiovascular and has protective effects on ischaemic cerebral apoplexy (Mou et al. 2017; Jung et al. 2018; Akter et al. 2019). The vinegar-processed Ezhu has been widely used for modulating the function of liver (Xu et al. 2018) and promoting blood circulation (Ayati et al. 2019; Lee et al. 2019). Studies (Lan et al. 2018; Cui et al. 2019) have confirmed that curcumin, elemene and curcumol have definite therapeutic effects on liver diseases through enhancing the function of the antioxidant defense system and inhibiting apoptosis of hepatocytes. During processing of astringent drugs, acidic components of vinegar tend to be free because of the action of acetic acid, so that both acidity and astringency are reinforced.

**Figure 3.**
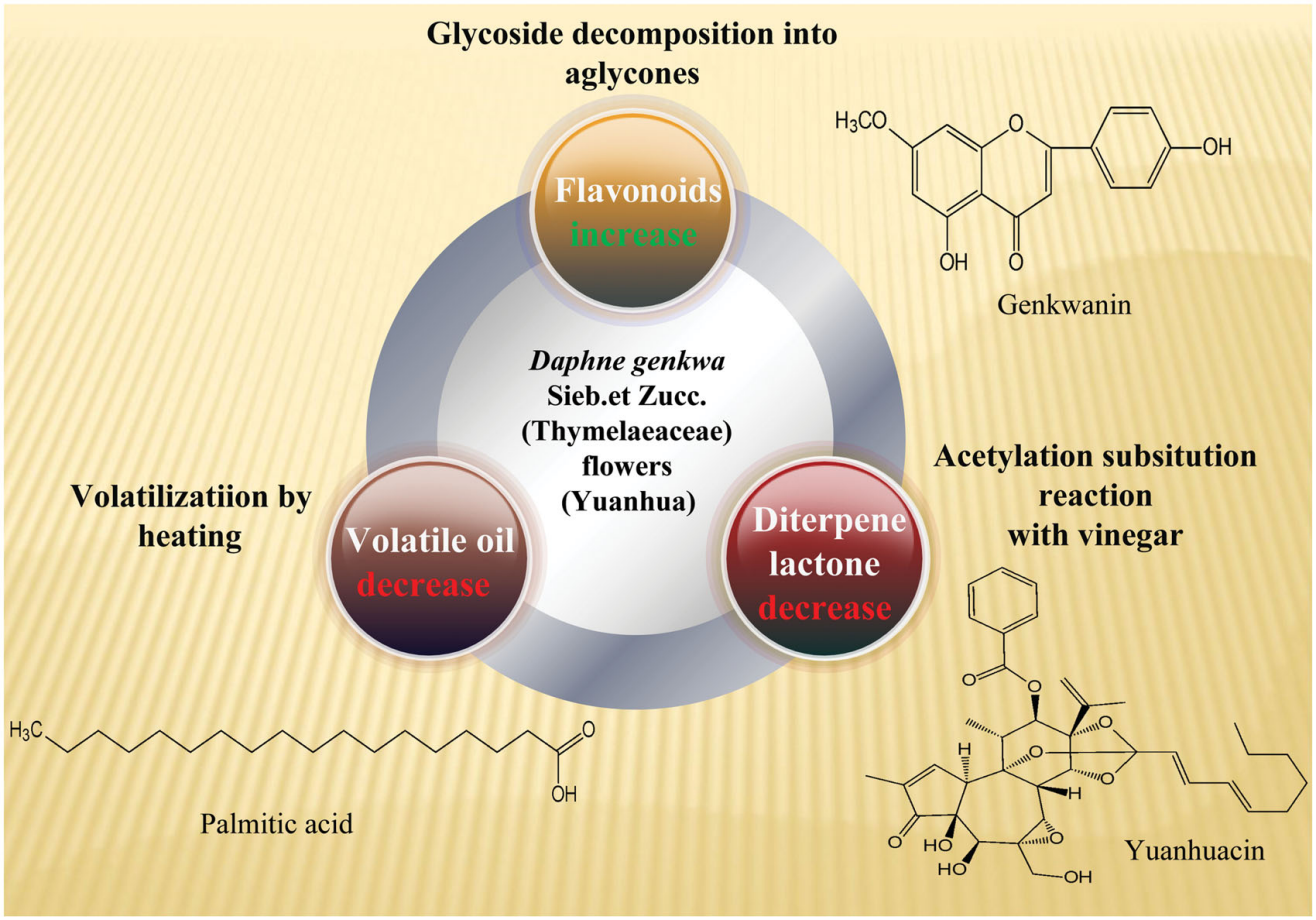
Efficacy enhancing and toxicity reducing of Yuanhua processing with rice vinegar.

Processing with vinegar can reduce drug toxicity and alleviate toxic and side effects of drugs. *Euphorbia kansui* T.N.Liou ex S.B.Ho. (Euphorbiaceae) tuberous root (Gansui) has been used as a traditional drug for the treatment of various diseases such as ascites, diabetes and leukaemia (Kim et al. 2017a). After processing of with vinegar, the content of twelve compounds that responsible for its toxic effect decreased which provided experimental evidence for the rational use (Zhang et al. 2018a). In terms of processing of toxic drugs, such as drastically purgating water drugs, for example, toxicity of *Daphne genkwa* Sieb. et Zuccc. (Thymelaeaceae) flowers (Yuanhua) reduces by changing chemical structure. Researchers found that vinegar-processing could enhance bioavailability of genkwanin, 3′-hydroxygenkwanin, apigenin and luteolin but reduce the bioavailability of yuanhuacine and genkwadaphnin (Tao et al. 2018). The efficacy enhancing and toxicity reducing of Yuanhua processing with yellow rice vinegar are shown in Figure 3.

Furthermore, vinegar processing can dispel smelly odours and change the physic-chemical properties of drugs for better clinical application. *Boswellia bhaurdajiana* Birdw. (Burseraceae) resin (Ruxiang) has a bad smell. Oral administration can cause vomiting easily. Processing with vinegar can dispel their smelly odours and reduce the side effects (Pan et al. 2015). A possible mechanism of dispelling smelly odours by processing with vinegar is that heating of drugs with vinegar can promote volatilization of the smell and the vinegar odour covers the fishy smell. Vinegar can also mask unpleasant odour and the aqueous extract could exert certain protective effect on ischemia-reperfusion injury (Zhai 2016). In addition, researchers (Liang et al. 2019) found that vinegar processing can alter the surface morphology, decrease the particle size and polydispersity index, raise the absolute value of the zeta potentia, specific surface area and porosity, and drop the viscosity of Ruxiang. Meanwhile, the rates of absorption and dissolution of the boswellic acid were increased after processing with vinegar. It accords with ‘toxicity reducing and efficacy enhancing’ in TCM and lays a foundation for modern research on TCM. Mineral drugs, such as magnetite and red ochre, are hard and difficult to decoct their active constituents out. Hence, they must be processed with vinegar before being used as medicine. Processing of mineral drugs and shell drugs with vinegar can make these drugs loose and fragile, promote the dissolution of active ingredients. For example, calcining and vinegar quenching can change crystal forms of mineral drugs, enhance solubility of these drugs and generate acetate, facilitating decoction and absorption.

Scholars have done a great deal of research on the mechanism of increasing the efficacy and reducing the toxicity of vinegar processing, but most of the research focuses on the changes of chemical composition, enhancing the efficacy and decreasing the toxicity before and after processing. There are few researches on the deep mechanism of causing these changes. It is necessary to make full use of modern scientific means to study the mechanism of vinegar processing such as metabolomics, molecular mechanism, etc. Moreover, vinegar is a complex body containing a variety of components and has certain pharmacological effects. But there are few studies on the components of vinegar. Therefore, it is necessary to consider the constituents and pharmacological actions of vinegar in the research on the mechanism of increasing the effect and reducing toxicity.

### Processing with honey

In TCM, honey has been widely used as a type of traditional Chinese medicine for more than two thousand years and as an additive in the processing of herbal medicines to enhance the immunostimulatory activities (Bogdanov et al. 2008; Fukuda et al. 2011). When used as an auxiliary material, honey must be heated. Heated honey reveals immunostimulatry and macrophase phagocytosis both *in vitro* and *in vivo* (Attia et al. 2008; Ota et al. 2019). According to Leigong Paozhi Lun, ‘drug processing with honey allows drugs become sweet and sluggish with effects of benefiting Qi, moistening lung, relieving a cough and stopping pain and dysentery’ (Lei 1986). According to Materia Medica Companion, ‘file velvet antler into crumbs, soak these crumbs in 5 L of whitish honey, decoct the mixture on low fire until it becomes extremely dry, pound and screen the product. The processed drug can tonify Qi, strengthen bone marrow and repair severe injury’ (Chen 1988). During the Ming Dynasty, people thought that ‘it is difficult to dissolve drugs processed with honey and these drugs can tonify primordial yang’. This method is usually used to process drugs with tonic and moisture effects.

*Astragalus membranceus* (Fisch.) Bge. (Lamiaceae) root is widely used throughout the world. Scientific evidence shows that Hungqi has multiple biological activities, including immune-modulatory, anti-inflammatory, antioxidant, renal protective and cardioprotective activities (Li et al. 2019). The enhancement of immune function is one of the main pharmacological effects. Honey-processed Huangqi as a widely used Qi-tonifying and immunomodulating herb in TCM, has strengthen the tonic effects and achieve fewer side effects compared with raw Huangqi in clinical application (Shi et al. 2015; Liu et al. 2018a). The addition of honey increased the concentrations of active compounds and their oral bioavailability, provided protective effect against acetylation, and consequently increased their bioactivity (Dai et al. 2020). By comparing the chemical composition of raw and honey-processed Huangqi, researchers (Huang et al. 2018) found that 20 compounds were differential components and 1 metabolite only existed in the honey processing group, which might provide the basis for explaining the biologic activity differences in the treatment of TCM. TCM theory says that tonic effect often associated with improved immune function (Wang et al. 2012). *Glycyrrhiza uralensis* Fisch. (Leguminosae) root and rhizome (Gancao) and honey-processed Gancao are two major specifications in clinical application. The immune function before and after processed with honey had obvious difference, which was enhanced after honey processing (Fu et al. 2014). Modern research (Zhang et al. 2018b) indicates that the absorption of isoliquiritin in rats was reduced while the absorption of isoliquiritigenin was promoted in the honey processing. Furthermore, the C-max of glycyrrhetinic acid was increased, suggesting that it may enhance the tonic effect of Gancao. These results may provide one explanation as to why honey-processed Gancao is better at invigorating Qi and restoring pulse. After processing with honey, the moisture effect of *Tussilago farfara* Linn. (Compsitea) flowers (Kuandonghua) and *Stemona japonica* (BI.) Miq. (Stemonaceae) tuber and root (Baibu) can be reinforced (Chen et al. 2013; Ling et al. 2013; Dong et al. 2016).

In addition, honey processing mitigates the bitter and bad properties of drugs. After processing of *Aristolochia debilis* Sieb. et Zucc. (Aristolochiaceae) fructus (Madouling) with honey, its bitter and cold properties damaging stomach are removed. Madouling has been popularly prescribed in China to treat a range of conditions including wound healing, arthritis and gynecological problems (Zhu et al. 2015). Aristolochic acids, the plant extract of *Aristolochia* spp, is considered as the main ingredient (Zhou et al. 2017; Liu et al. 2018b). Current evidence has demonstrated that aristolochic acids can cause mutagenicity, carcinogenicity and nephrotoxicity (Chen et al. 2006; Hoang et al. 2013; Tsai et al. 2013). The acute and subacute experiments (Yuan et al. 2014) showed a dose-dependent relationship of the nephrotoxicity of raw Madouling and honey-processed Madouling. But the toxicity growth rate of Madouling was bigger than that of honey-processed. Even when the contents of aristolochic acid were equivalent, the toxic effect of raw Madouling was more serious than that of honey-processed group. Some scholars think that the content of aristolochic acid in Madouling is decreased by stir-frying with honey, which shows the treatment of stir-frying with honey could reduce its toxicity (Liang et al. 2012). Further experiments (Yuan et al. 2017b) showed that five aristolochic acids shared a similar nonlinear PK (pharmacokinetic) process. They involve rapid absorption and elimination, and they were fit into a two-compartmental open model. Some significant pharmacokinetic differences were observed between raw Madouling and honey-processed Madouling: the C_max_ and AUC values of aristolochic acids I and aristolochic acids II in the raw Madouling groups were much higher than those of the honey-processed groups. Therefore, the honey processed Madouling is recommended in clinical use for its safety.

### Processing with saline water

Salt is one of the common condiments in daily life. The main chemical composition of salt is sodium chloride, and it also contains magnesium chloride, magnesium sulphate, calcium sulphate and other elements such as iron, phosphorus, iodine. Originally, it was recorded in Sheng Nong’s Herbal Classic as a middle-grade drug. It is salty in taste and cold in nature and belongs to stomach, kidney, large and small intestine meridians with effects of promoting vomiting, clearing heat, cooling blood and detoxifying. It is often used to treat food stopping in Shangwan, swelling pain of chest and abdomen, phlegm aggregation in chest, difficulty in urination and defaecation, haemorrhage of teeth and eyes, laryngalgia, dental fistula and swelling and ulcer on the body surface. Drug processing with salt water is one of the common methods of drug processing (Chen et al. 2018). So far, the pharmacopoeia and regional processing norms mainly include stir-frying with salt and steaming with salt etc. As for stir-frying with salt, saline water can be added before stir-frying or during stir-frying, where drugs are heated on slow fire. After stir-frying to a certain extent, drugs are taken out to cool. Saline water is often used to process drugs with effects of tonifying the kidney to arrest spontaneous emission and diuretic action.

Drug processing with salt can conduct the drug to the kidney meridian and strengthen the efficacy of drugs on lower-jiao diseases. The physiological characteristics of the kidney determine that it has a special reabsorption effect on Na^+^. In clinical application, a large number of active ingredients in salt-processed drugs are absorbed by the kidney along with the reabsorption of Na^+^. *Eucommia ulmoides* Oliver. (Eucommiaceae) cortex (Duzhong) is widely used as a botanical tonic in Asia as a traditional medicine plant and its bark can act as a diuretic, lower blood pressure, regulate the immune system, prevent osteoporosis and exhibit an anti-complement activity (Fang et al. 2019; Fu et al. 2019; Wang et al. 2019a). *Psoralea corylifolia* Linn. (Leguminosae) frucutus (Buguzhi) has been proved that they have many pharmacological effects including anticancer, anti-angiogenesis and promoting bone formation (Choi et al. 2016; Kim et al. 2017b). According to TCM theory, Duzhong and Buguzhi are both required to be salt-fried when they play a role in tonifying liver and kidney. It was reported that the salt-processed decoctions can enhance the efficacy and the enhancement may be due to the salt-frying affecting the absorption behaviour of the main components (Lu et al. 2018). Salt processing can promote the absorption and bioavailability of geniposide in Duzhong, even its absolute content decreased significantly after processing. The effect of inducing urination of *Plantago asiatica* Linn. (Plantaginaceae) seed (Cheqianzi) and *Alisma orientale* (Sam.) Juz. (Alismaceae) tuber (Zexie) can be strengthened through processing with salt. Modern pharmacology shows that Zexie has obvious diuretic effect and it can be used for the treatment of hypertension, chronic kidney failure, hyperlipidaemia and diabetes (Xiao et al. 2018). The diuretic effect of salt products was stronger than that of other processed products that may be related to the increase of alismol content in salt products (Cao et al. 2016; He et al. 2018). Moreover, processing with salt can promote anticorrosion of worm medicine.

### Processing with ginger juice

*Zingiber officinale* Roscoe. (Zingiberaceae) root and rhizome (ginger) a widely used herbal medicine, is warm and pungent according to TCM theory. To use ginger juice as medicine is common sense for people in China all the time. Ginger can prevent or arrest nausea and vomiting, inhibit the contraction of small intestine and protect the coronary artery (Thamlikitkul et al. 2017; Chatturong et al. 2018; Zhong et al. 2019b). During the Northern and Southern Dynasties, fresh ginger water started to be used as an adjuvant for drug processing in TCM. Fresh ginger juice can detoxify Banxia (Su et al. 2016), which has been extensively applied to clinic cases and it is often used in drug processing.

The main purpose of processing with ginger juice is to mitigate the cold nature and relieve the toxic and side effects of drugs. *Coptis chinensis* Franch. (Ranunculaceae) root (Huanglian) is cold and bitter in nature and can damage spleen yang easily, but the cold and bitter properties can be greatly removed by processing with ginger juice (Zhou et al. 2009). *Arisaema heterophy lum* Blume. (Aroideae) tuber (Tiannanxing) is a traditional Chinese herb used in the treatment of inflammation, convulsions and cancer (Kim et al. 2018). Because of its strong toxicity, it must be processed before application. It not only enhanced neuropharmacological efficacy but also reduced neurotoxic effects as compared to the raw decoctions (Huang et al. 2011). *Magnolia officinalis* Rehd. et Mills. (Magnoliaceae) cortex (Houpu) has long been used as a traditional Chinese medicine for the treatment of gastrointestinal disorders, anxiety, and bronchial asthma (Luo et al. 2019a). It is spicy in taste and can stimulate throat, which can be removed by processing with ginger juice (Zhao et al. 2018). In the current pharmacopoeia of various countries, the evaluation of the quality of commercial Houpo is effectively based on a quantitative determination of the levels of magnolol and honokiol (Wu et al. 2019). Processing with ginger juice consists of two parts: heating and ginger juice and both can affect the chemical composition of Houpu. The contents of magnolol and honokiol increased significantly after processing with ginger juice, while the contents of volatile oil were decreased obviously, which was considered to be consistent with the fact that ginger juice processing could attain the objective of treatment by efficacy enhancing and toxicity reducing.

Besides, processing with ginger can strengthen the effect of arresting vomiting of drugs. By processing of [*Bambusa tuldoides* Munro; *Sinocalmus beecheyanus* (Munro) McClure var. *pubescens* P. F. Li; *Phyllostachys nigra* (Lodd.) Munro var. *henonis* (Mitf.) Stapf ex Rendle middle layer of the stem] (Zhuru) with ginger juice, its effect of arresting vomiting is strengthened obviously. It is different from wine processing and vinegar processing mainly emphasizing the physical and chemical properties of liquid excipients, ginger juice processing restricted bias and enhanced curative effect by drug compatibility (Zhang et al. 2008).

### Processing with oil

Oil is often used to process drugs with effects of reinforcing the kidney yang and toxic drugs. Three methods of processing with oil are still widely used in clinical practice, including stir-frying with oil, frying with oil and steaming with oil. According to different types of oil and drugs used, the stir-frying method can be divided into several types, such as applying and stir-frying [*Cornus cervi* Pantotrichum. (Cervidae) horn (Lurong, Depei Bencao)], soaking and stir-frying [*Manis pentadactyla* Linnaeus. (Manidae) scute (Chuanshanjia, Zengguang Yanfang Xinpian)] and stir-frying with grease [*Epimedium brevicornum* Maxim. (Berberidaceae) blade (Yinyanghuo, Leigong Paozhi Lun)]. As for standards of stir-frying with oil, some drugs, such as *Stellera chamaejasme* Linn. (Thymelaeaceae) root (Langdu), Long-nosed pit viper Agkistrodon. (Crotalidae) body (Qishe), and *Gekko gecko* Linn. (Gekkonidae) body (Gejie), should be stir-fried to turn yellow; some drugs, such as Lurong and Tiger-bone, should be stir-fried to become yellow and crisp; and some drugs, such as antler, should be stir-fried to become fragrant. In history, drugs fried with oil include *Strychnos nux-vomica* Linn. (Loganiaceae) seed (Maqianzi), Tiger-bone, Chuanshanjia, *Whitmania pigra* Whitman. (Hirudinidae) body (Shuizhi), realgar, etc. Chuanshanjia and *Dendrobium nobile* Lindl. (Orchidaceae) stem (Shihu), were evenly mixed with oil and then steamed. In addition to the three methods above, there are methods of baking drugs applying oil [(*Scolopendra subspinipes*. (Scolopendridae) body (Wugong)] and lubrication with oil [*Citrus junos* Sieb.ex Tanaka. (Rutaceae) fructus (Zhiqiao)].

According to Bencao Mengquan, ‘after applying mutton fat and lard to drugs, drugs become fragile’. Thus, it can be seen that the primary purpose of processing with oil is to make drugs crisp and can be smashed easily. Most oils and fats used for drug processing are edible, sesame oil, mutton fat, butter and lard, for example. As most of them taste good and smell delicious, they can dispel smelly odours of some drugs, such as *Paratenodera sinensis* Saussure. (Mantidae) ootheca (Sangpiaoqiao).

Processing with oil can enhance the effect of reinforcing deficiency to assist Yang. Oil used for drug processing not only is non-toxic, but also has some therapeutic effects. Butter can tonify the five internal organs, benefit Qi and blood, quench thirst and moisten dryness; mutton fat can dispel cold-evil, benefit kidney and tonify yang; and lard can detoxify and benefit blood vessels. After processing, these oils can show a synergistic effect with drugs. The reinforced effects of tiger-bone after processing with mutton fat and leech after processing with lard may be closely related to the synergistic effect. Yinyanghuo has been used as tonic, antirheumatic and aphrodisiac herbs for bone loss, cardiovascular and impotence diseases for many years (Wang et al. 2019b; Kim and Shim 2019). It is usually fried with mutton fat, which could enhance the kidney invigorating and strengthen Yang. This method could increase the solubility of icariin and improve its intestinal absorption, so it should be one of the main processing methods in clinical to effectively improve the activity of Yinyanghuo (Chen et al. 2008; Cui et al. 2012; Zhang et al. 2018c). As representative component of processed Yinyanghuo, baohuoside I can form self-assembled m**i**celles under the action of sodium deoxycholate. Researchers (Gu et al. 2019) found that adding suet oil into the baohuoside I-bile salt micelles can make it form a more stable system, significantly improved encapsulation efficiency and drug loading, indicating that suet oil could improve the micelle formation significantly. In addition, the permeability coefficient of baohuoside-I increased and the oral bioavailability was also improved after adding the suet oil to the baohuoside-I bile salt micelles. All the results demonstrated that the oil can promote the formation and absorption of baohuoside-I self-assembled micelles, so as to enhance its synergistic effects.

### Processing with bile

Fresh bile of cows, pigs and sheep, which is usually brown green or dark brown liquid and mainly contains cholate, bile pigment and sterol, is used to process drugs. Bile is bitter in taste and cold in nature and can clear heat and relieve restlessness, benefit gallbladder and detoxicate, clear liver and improve vision.

After drug processing with bile, toxicity and dryness of drugs can be reduced and effects of clearing heat, resolving phlegm and relieving convulsion of drugs can be reinforced. Tiannanxing has been widely used over thousands of years in Asia, and its noticeable toxicity is documented in ancient books (Dong et al. 2015). According to TCM theory, after processing of Tiannanxing with bile, toxicity and dryness of the drug can be reduced or removed and its effects are transformed from eliminating dampness and resolving phlegm into clearing heat and resolving phlegm. From reducing swelling to arresting convulsion, where both properties and effects of the drug are changed. Modern research shown that, compared to the crude herbs, processed Tiannanxing (Dannanxing) not only enhanced neuropharmacological efficacy but also reduced neurotoxic effects (Huang et al. 2011).

### Processing with herbal juice

Herbal juice was first put forward in Leigong Paozhi Lun, where 91 processing methods with herbal juice were recorded in the book. This method is mainly applied to processing of toxic drugs to produce a synergistic effect, strengthen curative effects and reduce toxicity for safe medication. As processing excipients, such as Gancao juice and black soya bean juice belong to traditional Chinese medicine, chemical components, pharmacological effects and clinical indications of decoction pieces can be changed greatly after processing, increasing the difficulties in the study.

Gancao juice is the extract obtained by decocting Gancao and mainly composed of glycyrrhizin and liquiritin (Shan et al. 2018; Fan et al. 2019). It is sweet in taste and flat in nature and can tonify spleen and moisten lung, relieve spasm and pain, detoxify and harmonize drug effects. Processing with Gancao juice can moderates the nature of drugs, reduce their toxicity, harmonize drugs and reinforce the tonifying effects of drugs. The most recent report (He et al. 2019a) found the potential detoxification mechanism of liquorice by up-regulating efflux transporter as to reduce absorption of xenobiotics across small intestinal membrane. According to Mingyi Bielu, Gancao can detoxify various drugs. According to Jingyue Quanshu, Gancao can ease the acute property of *Aconitum carmichaelii* Debx. (Ranunculaceae) subroot (Fuzi) and detoxify drugs. Aconite has been extensively used in the treatment of rheumatoid arthritis, pain, inflammation, virus infection as well as for its cardiotonic actions, but it has highly toxic (Chou et al. 2018; Xu et al. 2019). Study shown that Gancao juice might lower the toxicity of aconite by reducing its exposure through inhibition of uptake transporters and may alleviate the toxicity caused by reduction of exposure through inhibition of uptake transporters (He et al. 2019b).

**Figure 4.**
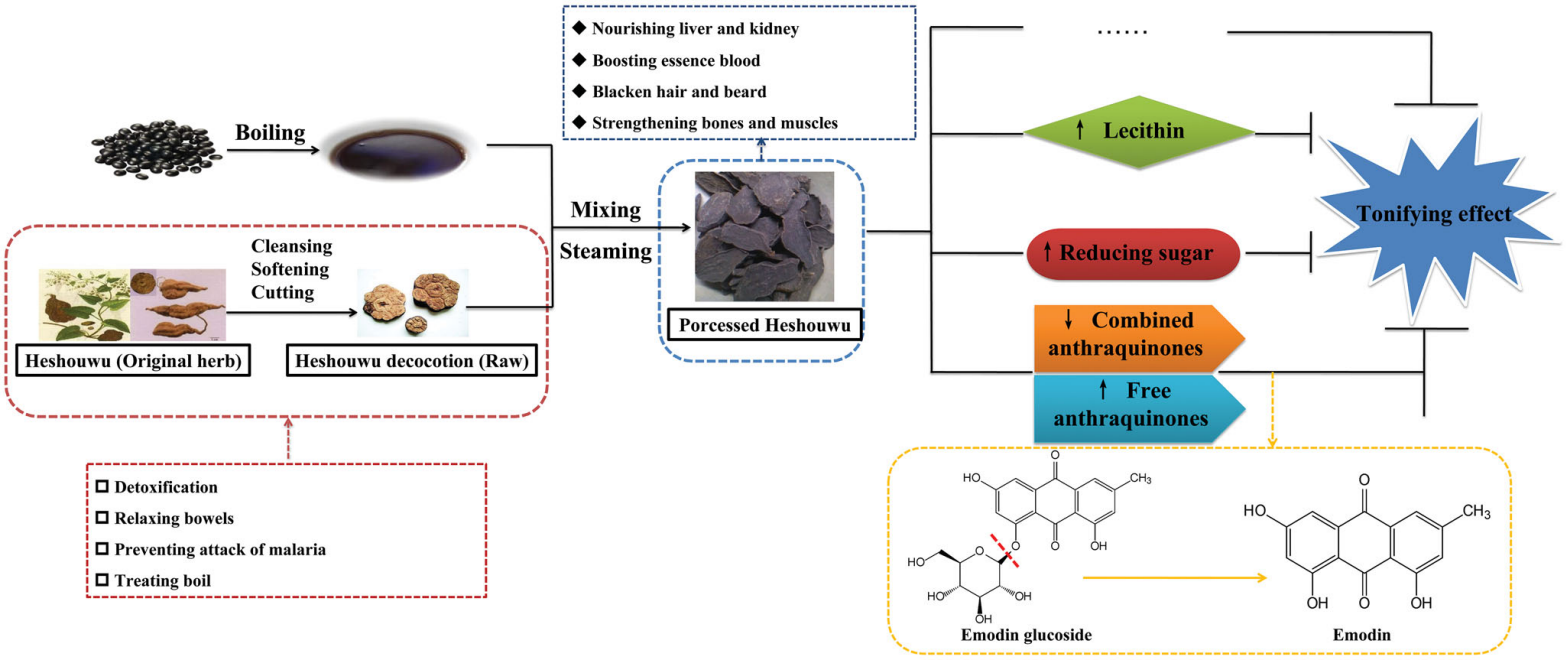
The process and purpose of Heshouwu processing with black soya bean juice.

The black soya bean juice refers to black turbid solution obtained by decocting black seeds of soybean and mainly contains protein, fat, saccharides, pigment, vitamins, starch and anthocyanins (Jhan et al. 2016). It is sweet in taste and flat in nature and can tonify liver and kidney, nourish the blood and promoted blood circulation, detoxicate and dispel the wind. Li Shizhen said, ‘five colours of beans corresponds to the five internal organs. Only black soya beans are cold in nature and can be used to treat kidney disease. It can remove swelling and wind-heat, invigorate the circulation of blood and detoxicate. If a person can often eat black soya beans, he or she will stay healthy’ (Li 1985). Drug processing with black soya bean juice can detoxicate drugs. Fuzi and Wutou are usually processed with black soya bean juice and can strengthen effects of tonifying liver and kidney of drugs. Raw Heshouwu is known to have carbuncle elimination, bowel relaxation, malaria prevention and detoxifying effects. Dianthrone derivatives are minor constituents and they are potential hepatotoxic components (Yang et al. 2019). Previous studies suggest that the processing method may affect the safety or efficacy of Heshouwu (Song et al. 2015). Steaming with black soya bean juice and drying by the sun shine nine times are the most traditional way to process Heshouwu. After processing, the effects of tonifying liver and kidney, benefiting essence and blood, blackening hair and strengthening muscles and bones of the drug are strengthened and the side effect of laxation is removed (Liu et al. 2018c; Luo et al. 2019b). The process and purpose of Heshouwu processing with black soya bean juice are shown in Figure 4.

Quality control is extremely important for the safe application of TCM. However, the quality control standards of TCM processing are weak. The technology standard of processing often depends on the operator’s experience which is strongly subjective and lacks objective criteria. For example, in Chinese Pharmacopoeia Commission (2015 edition), the technological steps of black soya bean juice only include decocting black soya bean twice, filtering, merging of filtrates and concentration without standard for chemical composition and quality. It is necessary to carry out the standard of liquid excipients, for it is as important as authentication to maintain the quality and safety of TCM.

## Summary and future prospects

Drug processing is a product of constant development of TCM and an important part of TCM treatment. In terms of drug processing with liquid excipients mainly including: wine, vinegar, ginger juice and honey, etc., which are common foods in daily life. The main aims of processing with liquid excipients are to enhance the efficacy and reduce the toxicity of drugs. Additionally, this method can moderate drastic action, modify the energetic properties diminish side effects, dissipate disagreeable odours and flavours, and so on. But liquid materials complicate the standardization of drug processing methods; thus, the traditional processing procedures need to be further organized, validated and implied with scientific standard to safeguard the quality of TCM (Zhao et al. 2010; Chen et al. 2018). As there are few studies on drug processing with these liquid excipients, many processing methods cannot be included into Chinese Pharmacopoeia Commission (2015). At present, these liquid excipients are primarily applied to the food industry. Although the processing has many advantages, there are still some problems.

### First, research on chemical composition and drug effect changes is not enough

At present, with the progress of analytical technology, the research on the chemical composition and efficacy changes of traditional Chinese medicine after processing is becoming more and more common, but it still needs to study deeper exploration. And less research on the changes of the active/toxic components in the body after processing, especially through excipients or other drugs after processing has been done.

### Second, research on processing principle lacks relevance

The research on the principle of processing mainly focuses on the change of chemical composition and pharmacological effect *in vitro*, and lacks the research on the relationship between the two changes. The role of liquid excipients in these changes is not fully explored.

### Third, liquid excipients lack scientific and uniform technology and standards

The lack of scientific and unified processing technology and standard of liquid excipient affects the quality and clinical application effect of TCM decoction pieces.

Taken together, in order to achieve better development, expand the source and application scope of traditional Chinese medicine, it is necessary to strengthen the in-depth study of modern science and solve the problems and factors that limit its development and application. We should attach great importance to standardized operation of processing of decoction pieces and strictly observe regulations relating to operation with practical spirit and be serious and cautious to ensure correct and reliable research results as well as good effects and safety of TCM treatment. Only in this way can TCM develop constantly to benefit more and more patients.
